# *PHACTR1* splicing isoforms and eQTLs in atherosclerosis-relevant human cells

**DOI:** 10.1186/s12881-018-0616-7

**Published:** 2018-06-08

**Authors:** Valérie-Anne Codina-Fauteux, Mélissa Beaudoin, Simon Lalonde, Ken Sin Lo, Guillaume Lettre

**Affiliations:** 1 Montreal Heart Institute, 5000 Bélanger Street, Montréal, Québec H1T 1C8 Canada; 2 Université de Montréal, 2900 Boul. Édouard-Montpetit, Montréal, Québec H3T 1J4 Canada

**Keywords:** *PHACTR1*, Alternative splicing, Expression quantitative trait locus (eQTL), Myocardial infarction (MI), Coronary artery disease (CAD)

## Abstract

**Background:**

Genome-wide association studies (GWAS) have identified a variant (rs9349379) at the phosphatase and actin regulator 1 (*PHACTR1*) locus that is associated with coronary artery disease (CAD). The same variant is also an expression quantitative trait locus (eQTL) for *PHACTR1* in human coronary arteries (hCA). Here, we sought to characterize *PHACTR1* splicing pattern in atherosclerosis-relevant human cells. We also explored how rs9349379 modulates the expression of the different *PHACTR1* splicing isoforms.

**Methods:**

We combined rapid amplification of cDNA ends (RACE) with next-generation long-read DNA sequencing to discover all *PHACTR1* transcripts in many human tissues and cell types. We measured *PHACTR1* transcripts by qPCR to identify transcript-specific eQTLs.

**Results:**

We confirmed a brain-specific long transcript, a short transcript expressed in monocytes and four intermediate transcripts that are different due to alternative splicing of two in-frame exons. In contrast to a previous report, we confirmed that the PHACTR1 protein is present in vascular smooth muscle cells. In 158 hCA from our collection and the GTEx dataset, rs9349379 was only associated with the expression levels of the intermediate *PHACTR1* transcripts.

**Conclusions:**

Our comprehensive transcriptomic profiling of *PHACTR1* indicates that this gene encodes six main transcripts. Five of them are expressed in hCA, where atherosclerotic plaques develop. In this tissue, genotypes at rs9349379 are associated with the expression of the intermediate transcripts, but not the immune-specific short transcript. This result suggests that rs9349379 may in part influence CAD by modulating the expression of intermediate *PHACTR1* transcripts in endothelial or vascular smooth muscle cells found in hCA.

**Electronic supplementary material:**

The online version of this article (10.1186/s12881-018-0616-7) contains supplementary material, which is available to authorized users.

## Background

Genome-wide association studies (GWAS) have identified a robust association between coronary artery disease (CAD) and the *PHACTR1* locus on chromosome 6p24 [1]. This locus is highly pleiotropic as the same allele associated with increased risk of CAD is protective of migraine [2], cervical artery dissection [3], and fibromuscular dysplasia [4]. Previously, we fine-mapped the CAD association to the candidate causal variant rs9349379 located in an intron of *PHACTR1* [5]. This SNP disrupts a MEF2 binding site and is an expression quantitative trait locus (eQTL) for *PHACTR1* in human coronary arteries (hCA) [5]. The eQTL result was replicated in the Genotype-Tissue Expression (GTEx) dataset [6]. *PHACTR1* encodes proteins of unclear biological functions: it was discovered in a yeast two-hybrid screen because it interacts with protein phosphatase-1 (PP1) and can also bind actin through its RPEL domains [7, 8]. Over-expression experiments have also implicated *PHACTR1* in apoptosis [9], angiogenesis [10], cellular matrix remodelling [11], and cell motility [8]. Although these processes are important for atherogenesis and CAD, the precise role(s) that PHACTR1 plays at the site of atherosclerotic lesions in coronary arteries is unknown.

Gupta et al. used CRISPR-Cas9 genome editing experiments in endothelial cells (ECs) derived in vitro from induced pluripotent stem cells to test the effect of genotypes at rs9349379 on the expression of nearby genes [12]. In this cellular system, the regulatory element around rs9349379 did not modulate the expression of *PHACTR1*, but controlled the expression of the endothelin-1 (*EDN1*) gene located 600 kb upstream. *EDN1* encodes ET-1, a 21 amino acids peptide with a potent vasoconstrictor effect in humans and clear implications for cardiovascular diseases. Paradoxically, rs9349379 is not an eQTL for *EDN1* in our hCA samples nor in the GTEx dataset [5, 6]. Together, these results raise the possibility that rs9349379 might control the expression of both *PHACTR1* and *EDN1* in different cell types and/or upon different environmental stimuli (e.g. inflammation).

For these reasons, we continued to investigate the regulation of *PHACTR1* expression in human tissues and cell types. Consistent with a recent study [13], we report here a long *PHACTR1* transcript expressed in the brain and a short transcript expressed in the brain, the heart, and monocytes. We also confirmed four intermediate transcripts that are different due to alternative splicing of two exons. They are co-expressed in tissues and cells tested. The shorter intermediate transcripts have a higher expression in the brain, the heart, and monocytes, whereas the longer intermediate transcripts are more expressed in ECs and vascular smooth muscle cells (VSMCs). Finally, we tested genetic associations between genotypes at rs9349379 and the expression of *PHACTR1* transcripts (or exons) in hCA samples. Our results strengthen the link between CAD-associated rs9349379 and *PHACTR1* expression in non-immune cells found in hCA.

## Methods

### Cell lines and tissues

We purchased pooled human umbilical vein endothelial cells (HUVEC; C2519A), human aortic endothelial cells (HAEC; CC-2535), and human coronary artery endothelial cells (HCAEC; CC-2585; Lonza) and cultured them in EBM-2 media supplemented with EGM-2 or EGM2-MV SingleQuot kit. We purchased immortalized HAEC (teloHAEC; CRL-4052; ATCC) and cultured them in vascular cell basal medium supplemented with vascular endothelial cell growth kit and puromycin at 0.3 μg/mL. Human coronary artery smooth muscle cells (HCASMC) and human aortic smooth muscle cells (HASMC) were obtained from Dr. Tardif’s lab and cultured in medium 231 (M-231-500) supplemented with smooth muscle growth supplement (S-007-25; Gibco). Monocytes were obtained from Dr. Rioux’s lab and we cultured them in RPMI with 10% fetal bovine serum. RNA from tissues was extracted either with the Ribopure Kit (Ambion) or using EZ1-XL Advance and Qiagen EZ1 RNA Tissue Mini Kit. hCA were obtained from the “Réseau d’Échanges de Tissus et d’Échantillons Biologiques” (RÉTEB) biorepository at the Montreal Heart Institute and Quebec Heart and Lung Institute. We purchased adult brain total RNA (540005–41, lot#6048990) and adult heart total RNA (540011–41, lot#6056165; Agilent Technologies).

### Rapid Amplification of cDNA Ends (RACE)

We extracted total RNA from HUVEC, teloHAEC, HCAEC, HAEC, HCASMC and HASMC P20 (with a passage (P) lower than 8 for primary cells unless specified) cells grown to confluence in 100 mm dishes with the RNeasy Plus Mini Kit (Qiagen). For the RACE experiments, we also added RNA from one hCA donor (#ICM167), adult brain, and adult heart. RNA integrity and concentration were measured by Agilent RNA 6000 Nano II assays (Agilent Technologies) on an Agilent 2100 Bioanalyzer. We reverse transcribed 1 μg of total RNA using a modified oligo (dT) primer and the SMARTScribe Reverse Transcriptase (Clontech). We specifically amplified cDNA of *PHACTR1* using the SMARTer RACE 5′/3’ Kit (Clontech). The gene specific primers are in Additional file 1. The RACE products were purified by gel extraction with the NucleoSpin Gel and PCR Clean-Up Kit, cloned in pRACE vector with the In-Fusion HD Cloning Kit and transformed with Stellar Competent Cells (Clontech). After overnight incubation, plasmids were extracted from single bacterial colonies with QIAprep Spin miniprep kit (Qiagen). We sequenced *PHACTR1* inserts by Sanger sequencing using M13 primers (Additional file 1).

### Long-read Pacific Biosciences (PacBio) sequencing library preparation

We produced libraries using 1 μg of total RNA (HUVEC, HAEC, HCAEC, HASMC, HCASMC and brain) and 1 μL of 12 μM dT-Barcode (dT-BC), according to the manufacturer’s protocol (Procedure and checklist-Isoform sequencing using the SMARTer PCR cDNA synthesis kit (Clontech) and no size selection). Each library had its own dT-BC (see Additional file 1 for sequences). Briefly, we synthesized the first strand of cDNA with the following thermal profile: 72 °C for 3 min, slow ramp to 42 °C at 0.1 °C/sec and 42 °C for 2 min. We synthesized cDNA using the SMARTer PCR cDNA synthesis kit at 42 °C for 90 min. We performed a large-scale PCR reaction on each cDNA library with optimization for the numbers of cycle using the KAPA Hi-Fi PCR kit. As determined with a High Sensitivity DNA Chip (Agilent Technologies), we needed 11 to 13 PCR cycles to obtain 250 ng of each cDNA. Before capture, we washed cDNA with AMPure PB beads. To enrich for *PHACTR1* transcripts, we designed biotinylated capture probes as Ultramers from IDT DNA according to the manufacturer’s protocol (full-length cDNA target sequence capture using SeqCap EZ libraries; see Additional file 1 for sequences). We used a total of 1.5 μg of cDNA (250 ng from each barcoded cDNA prep) to capture *PHACTR1* transcripts. We used six different blocking oligos, corresponding to the reverse complement sequence of the dT-BC with a 3′ modification (3SpC3)(Additional file 1). After capture, we amplified the library with 14 cycles of PCR using Takara LA Taq DNA polymerase (Clontech). The long-read PacBio sequencing was performed on one SMRTcell with Pacbio RS II at the Genome Quebec/McGill Innovation Center.

### Reverse transcription polymerase chain reaction (RT-PCR)

We extracted RNA from HUVEC, teloHAEC (treated with tumor necrosis factor (TNF)-α at 10 ng/ml for 4 h), monocytes, HCASMC, HASMC, HCAEC, and HAEC as described above. We analyzed unstimulated monocytes, as well as monocytes stimulated with 10 ng/ml of *phorbol myristate acetate* (PMA) for 48 h. Three hours later, some of the PMA-treated monocytes were also treated with lipopolysaccharides (LPS) (100 ng/ml) for one hour. We used the same adult brain total RNA and adult heart total RNA as for the RACE (see above). We measured RNA quality and concentration with Agilent RNA 6000 Nano II assays (Agilent Technologies) on an Agilent 2100 Bioanalyzer. We reverse transcribed 1 μg or less of total RNA (with RNA integrity number of 10 for all cells and above 7 for all tissues) using random primers and 1 U of the MultiScribe Reverse Transcriptase (Applied Biosystems) in a 20 μL reaction volume at 100 mM dNTPS and 20 U of RNase inhibitor with these three steps: 10 min at 25 °C, 120 min at 37 °C and 5 min at 85 °C. We mixed an equal volume of cDNA from each sample to create a pool of cDNA to use as positive control. We amplified fragments of *PHACTR1* transcripts from these tissues and cell lines with GoTaq DNA polymerase (Promega) with the following thermal cycle: 95 °C for 2 min; 94 °C for 30 s, optimal temperature for 30 s and 72 °C for 1 min and 20 s (35 times); 72 °C for 5 min. We also amplified full *PHACTR1* open reading frame (ORF) with SeqAmp DNA polymerase (Clontech) with the following thermal cycle: 94 °C for 1 min; 98 °C for 10 s, optimal temperature for 15 s and 68 °C for 3 min (30 times); 68 °C for 5 min. The sequences of the primers are in Additional file 1. We characterised the PCR products by agarose gel electrophoresis.

### Quantitative polymerase chain reaction (qPCR)

We extracted DNA and RNA from 36 hCA samples from the RÉTEB. Of these 36 samples, 14 are new and 22 were previously analyzed [5]. Genotyping of *PHACTR1*-rs9349379 was performed at the Beaulieu-Saucier Pharmacogenomics Centre of the Montreal Heart Institute on the Illumina Infinium MEGA_Consortium_v2 BeadChip: n_AA_ = 15, n_AG_ = 13, n_GG_ = 8. We measured RNA integrity and concentration with Agilent RNA 6000 Nano II assays (Agilent Technologies) on an Agilent 2100 Bioanalyzer. We reverse transcribed exactly 1 μg of total RNA as for the RT-PCR experiments (with RNA integrity number of 6 or above for all samples). We followed the MIQE guidelines to assess quality and reproducibility of our qPCR results [14]. We performed qPCR in triplicates for all samples using: 1.25 μL of cDNA (1/50 dilution), 5 μL of Platinum SYBR Green qPCR SuperMix-UDG (Life Technologies) and 3.75 μL of primer pair mix at 0.8 μM on a CFX384 from Biorad or Eco Illumina qPCR system (Montreal Biotech). We used the following thermal profile: 10 min at 95 °C, and 40 cycles of 30 s at 95 °C, 30 s at 55 °C and 45 s at 72 °C. We carried out melting curve analyses after the amplification process to ensure the specificity of the amplified products. We also simultaneously performed qPCR reactions with no template controls for each gene to test the absence of non-specific products. Cq values were determined with the CFX Manager 3.1 (Bio-Rad) software and expression levels were normalized on the expression levels of the house-keeping genes TATA-box binding protein (*TBP*), hypoxanthine-guanine phosphoribosyltransferase (*HPRT*), and glyceraldehyde 3-phosphate dehydrogenase (*GAPDH*) using the ΔΔCt method. Based on geNORM principles for accurate normalization of qPCR data by geometric averaging of multiple internal control genes [15], mean M values of 0.5845 and 0.5356 (for two experiments) were generated from the *GAPDH*, *HPRT1*, and *TBP* genes. The primer sequences are in Additional file 1.

### eQTL analyses using GTEx data

We downloaded from dbGaP genotypes at *PHACTR1*-rs9349379 and RNA-sequencing (RNA-seq) data from 122 hCA samples generated by the GTEx Project [6]. We used Trimmomatic (V0.36) on reads with the paired-end (PE) function to remove adapter sequences (TruSeq3-PE.fa:2:30:10) and to trim low-quality bases with the following parameters: LEADING:3 TRAILING:3 SLIDINGWINDOW:4:15 MINLEN:36. We then performed quality control checks using FastQC (V0.11.5). We aligned reads to the reference human genome sequence (GRCh37) using HISAT2 (V2.0.5) [16]. We selected reads that mapped to the *PHACTR1* locus using BamTools (V2.4.1) with the parameters -region “6:12717037..13288073” -mapQuality “> = 30”, and used SortSam and BuildBamIndex tools from Picard (V1.130) to convert .sam files into .bam files and to build their index files. We quantified *PHACTR1* expression with Stringtie (V1.3.3b) [16] with parameters -G -e -b; for these analyses we provided our own GTF file with genomic coordinates corresponding to the three *PHACTR1* transcripts expressed in hCA (Additional file 2). Finally, we used Ballgown (V2.8.4) [16] to extract exon-level expression data for eQTL analyses with genotypes at *PHACTR1*-rs9349379.

### Statistical analyses

We normalized *PHACTR1* expression data (for full transcripts or exons) from our qPCR experiments or GTEx using inverse normal transformation. Using linear regression in R, we tested the association between *PHACTR1* expression levels and genotypes at *PHACTR1*-rs9349379 (additive coding), correcting for sex, RNA integrity number (RIN), and the recruitment center (Montreal or Quebec city) when available.

### Western blot

To detect PHACTR1 protein expression in VSMCs, 0.75 × 10^6^ and 1 × 10^6^ of cryopreserved HASMC and HCASMC cells, respectively, were pelleted and directly lysed in RIPA buffer (50 mM Tris-HCl, pH 7.4, 1% NP-40, 0.25% Na-deoxycholate, 0.1% SDS, 150 mM NaCl) containing protease inhibitor cocktail (Sigma), phosphatase inhibitor cocktail 2 and 3 (Sigma), and 1 mM PMSF. As controls, teloHAEC were transfected with 20 nM of scramble (Santa Cruz, sc-37,007) or PHACTR1 (Santa Cruz, sc-95,456) small interfering RNA (siRNA) in 2 mL of siRNA transfection medium (Santa Cruz, sc-36,868) for 4 h using siRNA Transfection Reagent (Santa Cruz, sc-29,528), and then grown for 48 h before cell lysis and protein extraction. Homogenized lysates were clarified by centrifugation to eliminate insoluble cell debris. We determined protein concentration with a Pierce BCA Protein Assay Kit (ThermoFisher) and prepared samples containing 30 μg of proteins in a 600 mM DTT reducing buffer that was denatured 5 min at 95 °C prior to loading on a 8% polyacrylamide gel (running buffer: 25 mM Tris base; 192 mM glycine; 0.1% SDS). We transferred proteins onto nitrocellulose membranes in Towbin buffer (25 mM Tris base; 192 mM glycine, 20% (*v*/v) methanol) and incubated them for 1.5 h in the blocking buffer (1× TBS, 0.1% Tween-20, 5% milk) at room temperature. We then incubated membranes with primary antibodies against PHACTR1 (1:1000; custom antibody generated by Biomatik) or GAPDH (1:10,000; Cell Signaling, #2118) at 4 °C overnight. For the secondary antibody, we incubated membranes with horseradish peroxidase-conjugated anti-rabbit IgG (1:10,000; GE Healthcare, NA934) at room temperature for 1 h. The immunoblotting signal was revealed with the SuperSignal west Pico PLUS chemiluminescent substrate (Thermo scientific, 34,580) and visualized with the ChemiDoc Touch system (Bio-Rad).

## Results

### Six *PHACTR1* transcripts in human cells and tissues

**Fig. 1:**
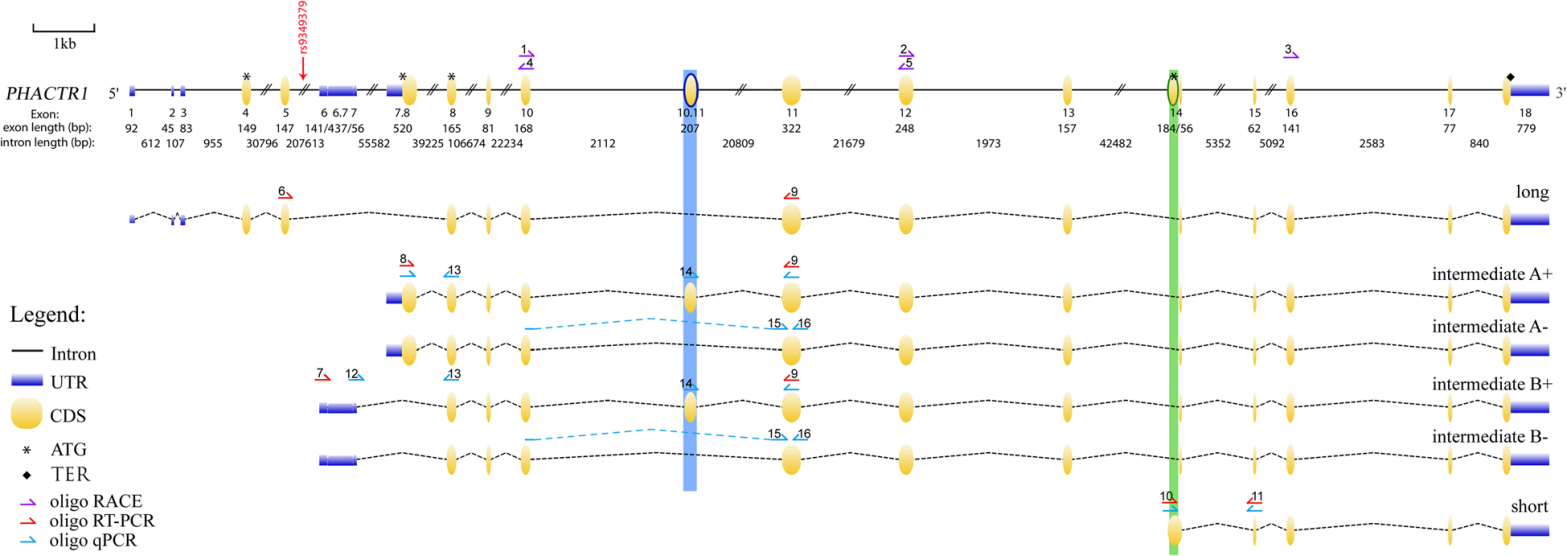
Six *PHACTR1* transcripts are expressed in human samples. We combined Rapid Amplification of cDNA Ends (RACE) and long-read DNA sequencing (Pacific Biosciences) to identify all *PHACTR1* mRNA transcripts expressed in human samples. Although we identified multiple transcripts, they give rise to six main transcripts. The schematic diagram shows the *PHACTR1* gene (top) and the six main transcripts below. The coronary artery disease-associated SNP rs9349379 (red arrow) is located in a large intron (207,613-bp) between exons 5 and 6. The start codons for the long, intermediate A, intermediate B and short transcripts are located, respectively, in exons 4, 7.8, 8, and 14. The stop codon for all six transcripts is in exon 18. In blue, we highlight the in-frame, 207-bp long, exon 10.11 that is included in intermediate transcripts A+ and B+. In green, we highlight part of exon 14 that is specific to the short transcript. We also indicated the different oligonucleotides used in our experiments; numbers referred to their sequences in Additional file 1. UTR, untranslated transcribed region; CDS, coding DNA sequence; ATG, start codon; TER, terminator codon. The figure was drawn to scale using GSDS2.0 [19]

Public databases (e.g. ENSEMBL, Gencode, GTEx) report > 10 different *PHACTR1* mRNA transcripts expressed in human samples. To confirm these results and comprehensively characterize the different *PHACTR1* transcripts expressed in humans, we combined RACE and long-read next-generation (PacBio) DNA sequencing experiments in many cell types and tissues. These two complementary approaches generated highly concordant results that we also validated by sequencing of the whole ORF. Although we did identify a large number of *PHACTR1* transcripts due to alternative splicing of non-coding 5′ and 3′ untranslated exons (Additional file 3), our transcriptomic profiling indicates that *PHACTR1* can give rise to six main different transcripts that would encode six proteins based on their coding sequences (Fig. 1and Additional file 4). The long transcript (1743-bp) encodes a protein of 580 amino acids (Fig. 1). We confirmed four intermediate transcripts that are different due to the inclusion of the alternatively spliced exons 7.8 and 10.11 (Fig. 1). Both alternative exons are in-frame and add respectively 279- (exon 7.8) and 207-bp (exon 10.11) to the ORF. Intermediate transcripts A+ (1953-bp) and B+ (1674-bp) include exon 10.11 and encode proteins of 650 and 557 amino acids. Intermediate transcripts A- (1746-bp) and B- (1467-bp) exclude exon 10.11 and encode proteins of 581 and 488 amino acids. Finally, we confirmed a short transcript (435-bp) encoding a protein of 144 aa (Fig. 1). Its start codon is located in exon 14, 83-bp upstream of the 3′ splice site for the long and intermediate transcripts (Additional file 5).

### *PHACTR1* transcripts are differentially expressed

**Fig. 2:**
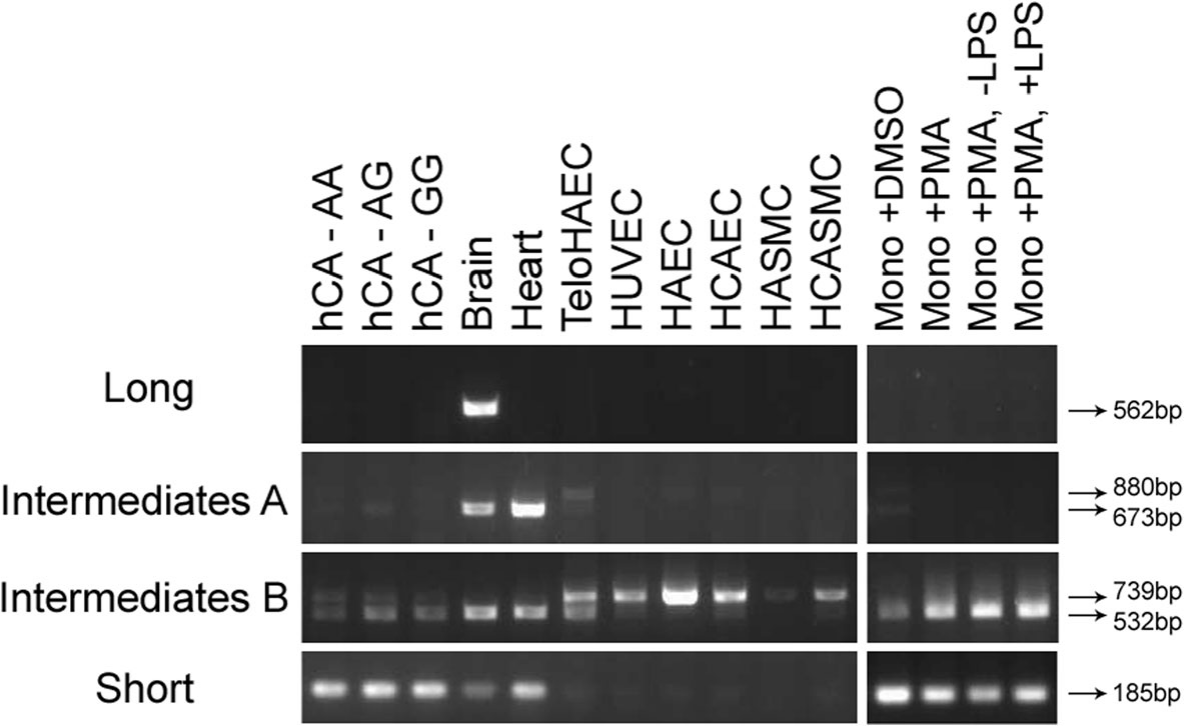
*PHACTR1* transcripts differential expression in human tissues and cell types. We designed transcript-specific primers and amplified *PHACTR1* by RT-PCR. The PCR fragment for the long transcript is 562-bp. We did not amplify a 769-bp fragment using this set of primers, indicating that exon 10.11 is absent from the long transcript. The PCR fragments for the intermediate transcripts A+, A-, B+ and B- are 880-bp, 673-bp, 739-bp and 532-bp, respectively. The PCR fragment for the short transcript is 185-bp. HUVEC: human umbilical vein endothelial cell; teloHAEC: immortalized human aortic endothelial cells; HCASMC: human coronary artery smooth muscle cells; HAEC: human aortic endothelial cells; HCAEC: human coronary artery endothelial cells; HASMC: human aortic smooth muscle cells; Mono: monocytes; PMA: phorbol myristate acetate; LPS: lipopolysaccharide

We used the sequence of the six *PHACTR1* transcripts to design transcript-specific pairs of primers and carried out RT-PCR experiments on mRNA extracted from human brain, heart, monocytes, ECs, and VSMCs (Fig. 2). The long transcript, which never included exons 7.8 nor 10.11, was only expressed in the brain [7]. In contrast, both intermediate transcripts B were expressed in all samples tested, although we noted that the inclusion of exon 10.11 varied from one sample to the other. Intermediate transcript B+ was more abundant in ECs and VSMCs, whereas intermediate transcript B- was the major intermediate transcript expressed in the brain, the heart, and in monocytes (Fig. 2, third panel from top). Both intermediate transcripts A were expressed in the brain, the heart, untreated monocytes, and ECs. Similar to what we saw for the intermediate transcripts B, the inclusion of exon 10.11 in the intermediate transcripts A was variable from one tissue to the other (Fig. 2, second panel from top). We validated that the short transcript was expressed in monocytes [13]. Stimulation with PMA or LPS to differentiate monocytes into macrophages, which are of interest regarding atherosclerosis, did not induce the expression of the long transcript, but seems to weakly increase the expression of intermediate transcripts B (Fig. 2). We detected expression of the short transcript in the heart and the brain, although this is consistent with the presence of immune cells within these tissues.

**Fig. 3:**
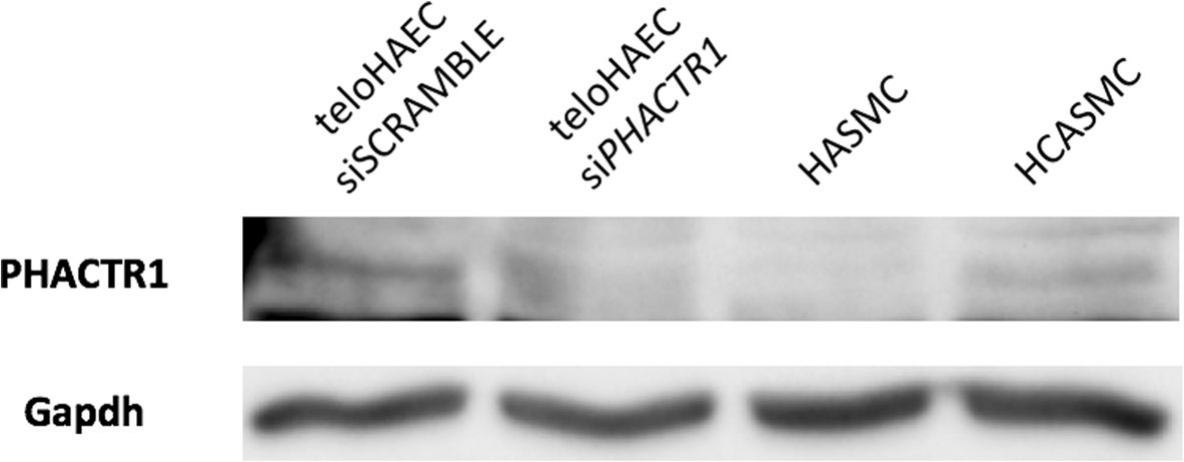
Presence of PHACTR1 protein in vascular smooth muscle cells. We tested for the presence of the PHACTR1 protein by immunoblotting using a custom anti-PHACTR1 antibody. The first and second lane, using teloHAEC protein extracts treated or not with a short interfering RNA (siRNA) against *PHACTR1*, serve as control to demonstrate the specificity of this anti-PHACTR1 antibody. Although we did not detect PHACTR1 in human aortic smooth muscle cells (HASMC), there is weak but specific band corresponding to PHACTR1 in human coronary artery smooth muscle cells (HCASMC). The expected 64 kDa PHACTR1 protein migrated with an apparent molecular weight of 74 kDa. GAPDH was used as loading control

Although *PHACTR1* transcripts had previously been detected in VSMCs, immunoblotting with a commercially available anti-PHACTR1 antibody failed to detect the corresponding protein [13]. We purchased and tested all commercially available anti-PHACTR1 antibodies, but none showed specificity in short interfering RNA (siRNA)-mediated knockdown experiments. For this reason, we developed our own polyclonal anti-PHACTR1 antibody (**Materials and methods**) and have shown its specificity using siRNA against *PHACTR1* [17]. Using this antibody, we detected weak PHACTR1 protein expression in VSMCs from hCA (Fig. 3).

### CAD-associated rs9349379 is an eQTL for *PHACTR1* intermediate transcripts A

Using primers that capture the long and intermediate *PHACTR1* transcripts, we previously showed that genotypes at rs9349379 are associated with *PHACTR1* expression levels in hCA [5]. That result was subsequently replicated in the GTEx database. Given that hCA are composed of different cell types (ECs, VSMCs, and immune cells) and that *PHACTR1* transcripts are differentially expressed across tissues and cell types, we tested if rs9349379 is associated with the levels of all or specific *PHACTR1* transcripts. We obtained DNA and RNA from 36 hCA, including 14 new samples not analyzed in our previous study [5]. We genotyped rs9349379 and measured by qPCR the levels of each of the five *PHACTR1* transcripts expressed in hCA. We found a significant association between rs9349379 and intermediate transcripts A+ and B+, whereas the SNP was not significantly associated with intermediate transcripts A- and B- (Table 1 and Additional file 6). Although we detected strong expression of the short *PHACTR1* transcript in hCA, genotypes at rs9349379 were not associated with its levels (Table 1 and Additional file 6).

**Table 1:** rs9349379 is an eQTL for *PHACTR1* intermediate transcripts A+ and B+ in human coronary arteries (hCA)

Transcripts	Beta (SE)	*P*-value	Variance explained (%)
Intermediates A+ and A-	−0.49 (0.21)	0.026	7.8
Intermediates B+ and B-	−0.54 (0.17)	0.0040	17.5
Intermediates A+ and B+	−0.56 (0.19)	0.0073	16.4
Intermediates A- and B-	−0.35 (0.21)	0.10	–
Short	−0.068 (0.20)	0.73	–

We tested by linear regression the association between genotypes at rs9349379 (additive model) and normalized expression levels for the different *PHACTR1* transcripts expressed in hCA. The long *PHACTR1* transcript is not expressed in hCA. Effect sizes (Beta) and standard errors (SE) are in standard deviation units. The direction of the Beta is for the G-allele. We analyzed 36 hCA samples (N_AA_ = 15, N_AG_ = 13, N_GG_ = 8). The frequency of the G-allele was 40%. The G-allele, associated with increased coronary artery disease risk, is associated with reduced *PHACTR1* expression. When significant (*P*-value < 0.05), we provided the percentage of *PHACTR1* expression variation explained by genotypes at rs9349379

Because our hCA sample size remains small, we attempted to replicate these eQTL results in the larger GTEx dataset with genotype and gene expression data available from 122 hCA. Since it is computationally challenging to assemble specific mRNA transcripts out of short-read RNA-seq results, we opted to test the association between rs9349379 and the expression levels of specific *PHACTR1* exons. Overall, we found nominally significant associations (*P*-value < 0.05) with exons common to the intermediate and short transcripts (Table 2). However, the association between rs9349379 and the expression levels of the 5′ end of exon 14 that is specific to the short transcript (Additional file 5) was not significant (*P*-value = 0.47) (Table 2 and Additional file 6). Thus, using qPCR and RNA-seq gene expression data, we could not detect an eQTL effect between rs9349379 and the expression of the immune-specific short *PHACTR1* transcript in 158 hCA samples.

**Table 2:** Replication of rs9349379 eQTL effects in human coronary arteries (hCA) from GTEx

Exon	Exon length (bp)	Beta (SE)	*P*-value	Variance explained (%)
6 (5’UTR)	141	−0.084 (0.13)	0.51	–
7 (5’UTR)	56	−0.11 (0.13)	0.39	–
7.8	520	−0.15 (0.12)	0.22	–
8	165	−0.31 (0.13)	0.017	3.4
9	81	−0.19 (0.13)	0.14	–
10	168	−0.35 (0.13)	0.0065	3.1
10.11	207	−0.30 (0.13)	0.022	3.9
11	322	−0.35 (0.13)	0.0074	2.8
12	248	−0.40 (0.13)	0.0019	5.0
13	157	−0.28 (0.13)	0.029	3.0
14^a^	240	−0.17 (0.13)	0.20	–
5′-14^b^	184	0.095 (0.13)	0.47	–
3′-14^c^	56	−0.25 (0.13)	0.059	–
15	62	−0.25 (0.13)	0.058	–
16	141	−0.27 (0.13)	0.042	2.3
17	77	−0.31 (0.13)	0.017	3.5
18 (3’UTR)	779	−0.25 (0.13)	0.055	–

We analysed by linear regression the association between genotypes at rs9349379 (additive model) and normalized levels of *PHACTR1* exon expression. Expression levels were corrected for the length of the exons (**Materials and methods**). Effect sizes (Beta) and standard errors (SE) are in standard deviation units. The direction of the effect size is for the G-allele, associated with increased coronary artery disease risk. We analyzed data from 122 hCA samples (N_AA_ = 48, N_AG_ = 57, N_GG_ = 17). The frequency of the G-allele was 37%. When significant (*P*-value < 0.05), we provided the percentage of *PHACTR1* exon expression variation explained by genotypes at rs9349379. ^a^The full exon 14; ^b^part of exon 14 which is specific to the short *PHACTR1* transcript; ^c^part of exon 14 which is present in all *PHACTR1* transcripts (see Additional file 5 for details)

## Discussion

Despite the recent report linking *PHACTR1*-rs9349379 with *EDN1* in ECs [12], *PHACTR1* itself remains a strong causal candidate gene at this CAD-associated locus because of the eQTL effect in hCA [5, 6]. Further, it was recently shown in an in vitro cell culture system that PHACTR1 contributes to VSMC calcification, a hallmark of CAD [18]. Using two different methods, we confirmed that the human *PHACTR1* gene encodes six main transcripts that are differentially expressed. Although our results are largely consistent with a study by Reschen et al. [13], we note three important differences: First, we confirmed that there are six, and not three, *PHACTR1* transcripts. Indeed, whereas Reschen and colleagues reported only one intermediate transcript (transcript B+ in Fig. 1), we found that four intermediate transcripts (with or without exons 7.8 and 10.11) are co-expressed in the heart (Fig. 2). Whether these four PHACTR1 isoforms, of 650, 581, 557 and 488 amino acids, have the same functions is currently unknown. Second, we confirmed using our own custom polyclonal antibody against PHACTR1 that the protein is present in VSMCs. In contrast, we have not been able to detect by immunoblotting the protein encoded by the short transcript. And third, our eQTL analyses in 158 hCA validated an effect of genotypes at rs9349379 on the expression of intermediate transcripts A+ and B+, but not the short immune-specific transcript. One possible interpretation of this result is that rs9349379 mediates in part its effect on CAD risk by regulating the expression or splicing of these *PHACTR1* intermediate transcripts in ECs and/or VSMCs within hCA, and not through the short immune-specific *PHACTR1* transcript. In support of this hypothesis, we also know that rs9349379 is associated with fibromuscular dysplasia [4], a disease without an inflammatory component. Precisely how, at the molecular level, genotypes at rs9349379 modulate cell type-specific transcriptional expression and/or splicing remains to be determined.

Our study presents with some limitations. As for every transcriptomic profiling experiments, weakly expressed transcripts might have missed detection. We tried to account for this by combining two complementary methods and profiling *PHACTR1* transcripts in high quality mRNA prepared from many tissues and cell types. The number of available hCA samples for our eQTL analyses was small (*N* = 36). To increase statistical power, we also analyzed RNA-seq data from the GTEx Project (*N* = 122). Because short-reads cannot be used unambiguously to reconstruct specific transcripts, we decided to analyze the expression levels of specific exons. This analysis generated results that were highly concordant with our transcript-specific qPCR results. Finally, although we did not detect an eQTL effect for the short *PHACTR1* transcript in hCA, it does not rule out a potential role for *PHACTR1* in immune cells in the context of atherosclerosis progression and CAD. Indeed, the short *PHACTR1* protein might play a critical atherosclerotic function in circulating monocytes, which would not have been captured in hCA samples. However, it is critical to confirm that the short *PHACTR1* transcript can be translated into an active protein in human cells.

## Conclusions

In this article, we provide a comprehensive survey of *PHACTR1* transcripts in tissues and cells that are relevant to atherosclerosis initiation and progression. Importantly, we confirmed that the PHACTR1 protein is present in VSMCs and that the CAD-associated variant rs9349379 associates with the expression of specific *PHACTR1* transcript in hCA. Gupta and colleagues have argued that the molecular link they identified between rs9349379 and *EDN1* expression largely accounts for the association of this locus with several vascular diseases [12]. One natural extension of this model, which would be consistent with the pleiotropy observed as well as the *PHACTR1* eQTL we detected in hCA, is that the same SNP influences the expression of two genes – *EDN1* and *PHACTR1* – which are expressed in CAD-relevant cells and could act together to contribute to vascular disease risk.

## Additional files

Additional file 1: List of primers used in this study. The numbers in the “Name” column refers to the primers shown in Fig. 1. All sequences are given in the 5′ to 3′ orientation. (PDF 43 kb)

Additional file 2: Genomic coordinates of exons based on the reference human genome sequence GRCh37/hg19. (PDF 47 kb)

Additional file 3: Summary of observed transcripts with their alternatively spliced 5′ and 3′ untranslated regions (UTRs). The blue rectangles on the left indicate transcripts that were detected in tissue samples whereas gray rectangles mean they were not detected. Introns are not shown for simplicity; black lines indicate that the exons were not present in the transcript. (PDF 75 kb)

Additional file 4: Transcript and protein IDs of *PHACTR1* gene (221,692; ENSG00000112137). (PDF 45 kb)

Additional file 5: Zoom-in representation of *PHACTR1* exon 14. The 5′-14 fraction of exon 14 is specific to the short immune-specific *PHACTR1* transcript. We also illustrate the 3′ splice site used for the long and intermediate *PHACTR1* transcripts. In blue is the intron between exons 13 and 14. The red arrow indicates a primer used to detect the short *PHACTR1* transcript. The sequence of this primer #10 is in Additional file 1. (PDF 193 kb)

Additional file 6: Associations between genotypes at rs9349379 and *PHACTR1* expression levels in human coronary arteries. (**A**) By qPCR using transcript-specific primers, we measured the expression of *PHACTR1* transcripts in 36 human coronary arteries (hCA)(N_AA_ = 15, N_AG_ = 13, N_GG_ = 8). The long *PHACTR1* transcript is not expressed in hCA. (**B**) We used GTEx data to test the associations between rs9349379 and the expression levels of *PHACTR1* exons in 122 hCA (N_AA_ = 48, N_AG_ = 57, N_GG_ = 17). Here, we only show results for five exons, but association results for all *PHACTR1* exons are available in Table 2. Exon 10.11 corresponds to the alternatively spliced exon located between exons 10 and 11. 5′ exon 14 corresponds to part of exon 14 that is specific to the short *PHACTR1* transcript. (PDF 350 kb)

## Abbreviations

CAD: Coronary artery disease
dT-BC: dT-Barcode
ECs: Endothelial cells
*EDN1*: Endothelin-1
eQTL: Expression quantitative trait locus
*GAPDH*: Glyceraldehyde 3-phosphate dehydrogenase
GTEx: Genotype-Tissue Expression
GWAS: Genome-wide association studies
HAEC: Human aortic endothelial cells
HASMC: Human aortic smooth muscle cells
hCA: Human coronary arteries
HCAEC: Human coronary artery endothelial cells
HCASMC: Human coronary artery smooth muscle cells
*HPRT*: Hypoxanthine-guanine phosphoribosyltransferase
HUVEC: Human umbilical vein endothelial cells
LPS: Lipopolysaccharides
ORF: Open reading frame
PacBio: Long-read Pacific Biosciences
*PHACTR1*: Phosphatase and actin regulator 1
PMA: *Phorbol myristate acetate*
PP1: Protein phosphatase-1
qPCR: Quantitative polymerase chain reaction
RACE: Rapid amplification of cDNA ends
RÉTEB: Réseau d’Échanges de Tissus et d’Échantillons Biologiques
RIN: RNA integrity number
RT-PCR: Reverse transcription polymerase chain reaction
TBP: TATA-box binding protein
teloHAEC: Immortalized HAEC
TNFα: Tumor necrosis factor α
VSMCs: Vascular smooth muscle cells

## References

[1] Kathiresan S, Voight BF, Purcell S, Musunuru K, Ardissino D, Mannucci PM, Anand S, Engert JC, Samani NJ, Schunkert H (2009). Genome-wide association of early-onset myocardial infarction with single nucleotide polymorphisms and copy number variants. Nat Genet.

[2] Freilinger T, Anttila V, de Vries B, Malik R, Kallela M, Terwindt GM, Pozo-Rosich P, Winsvold B, Nyholt DR, van Oosterhout WP (2012). Genome-wide association analysis identifies susceptibility loci for migraine without aura. Nat Genet.

[3] Debette S, Kamatani Y, Metso TM, Kloss M, Chauhan G, Engelter ST, Pezzini A, Thijs V, Markus HS, Dichgans M (2015). Common variation in PHACTR1 is associated with susceptibility to cervical artery dissection. Nat Genet.

[4] Kiando SR, Tucker NR, Castro-Vega LJ, Katz A, D'Escamard V, Treard C, Fraher D, Albuisson J, Kadian-Dodov D, Ye Z (2016). PHACTR1 is a genetic susceptibility locus for fibromuscular dysplasia supporting its complex genetic pattern of inheritance. PLoS Genet.

[5] Beaudoin M, Gupta RM, Won HH, Lo KS, Do R, Henderson CA, Lavoie-St-Amour C, Langlois S, Rivas D, Lehoux S (2015). Myocardial infarction-associated SNP at 6p24 interferes with MEF2 binding and associates with PHACTR1 expression levels in human coronary arteries. Arterioscler Thromb Vasc Biol.

[6] Consortium GT (2015). Human genomics. The genotype-tissue expression (GTEx) pilot analysis: multitissue gene regulation in humans. Science.

[7] Allen PB, Greenfield AT, Svenningsson P, Haspeslagh DC, Greengard P (2004). Phactrs 1-4: a family of protein phosphatase 1 and actin regulatory proteins. Proc Natl Acad Sci U S A.

[8] Wiezlak M, Diring J, Abella J, Mouilleron S, Way M, McDonald NQ, Treisman R (2012). G-actin regulates the shuttling and PP1 binding of the RPEL protein Phactr1 to control actomyosin assembly. J Cell Sci.

[9] Jarray R, Allain B, Borriello L, Biard D, Loukaci A, Larghero J, Hadj-Slimane R, Garbay C, Lepelletier Y, Raynaud F (2011). Depletion of the novel protein PHACTR-1 from human endothelial cells abolishes tube formation and induces cell death receptor apoptosis. Biochimie.

[10] Allain B, Jarray R, Borriello L, Leforban B, Dufour S, Liu WQ, Pamonsinlapatham P, Bianco S, Larghero J, Hadj-Slimane R (2012). Neuropilin-1 regulates a new VEGF-induced gene, Phactr-1, which controls tubulogenesis and modulates lamellipodial dynamics in human endothelial cells. Cell Signal.

[11] Jarray R, Pavoni S, Borriello L, Allain B, Lopez N, Bianco S, Liu W, Biard D, Demange L, Hermine O (2015). Disruption of phactr-1 pathway triggers pro-inflammatory and pro-atherogenic factors: new insights in atherosclerosis development. Biochimie.

[12] Gupta R, Hadaya J, Trehan A, Zekavat S, Roselli C, Klarin D, Emdin C, Hilvering C, Bianchi V, Mueller C (2017). A genetic variant associated with five vascular diseases is a distal regulator of Endothelin-1 gene expression. Cell.

[13] Reschen ME, Lin D, Chalisey A, Soilleux EJ, O'Callaghan CA (2016). Genetic and environmental risk factors for atherosclerosis regulate transcription of phosphatase and actin regulating gene PHACTR1. Atherosclerosis.

[14] Bustin SA, Benes V, Garson JA, Hellemans J, Huggett J, Kubista M, Mueller R, Nolan T, Pfaffl MW, Shipley GL (2009). The MIQE guidelines: minimum information for publication of quantitative real-time PCR experiments. Clin Chem.

[15] Vandesompele J, De Preter K, Pattyn F, Poppe B, Van Roy N, De Paepe A, Speleman F (2002). Accurate normalization of real-time quantitative RT-PCR data by geometric averaging of multiple internal control genes. Genome Biol.

[16] Pertea M, Kim D, Pertea GM, Leek JT, Salzberg SL (2016). Transcript-level expression analysis of RNA-seq experiments with HISAT, StringTie and Ballgown. Nat Protocols.

[17] Lalonde S, Stone OA, Lessard S, Lavertu A, Desjardins J, Beaudoin M, Rivas M, Stainier DYR, Lettre G (2017). Frameshift indels introduced by genome editing can lead to in-frame exon skipping. PLoS One.

[18] Aherrahrou R, Aherrahrou Z, Schunkert H, Erdmann J (2017). Coronary artery disease associated gene Phactr1 modulates severity of vascular calcification in vitro. Biochem Biophys Res Commun.

[19] Hu B, Jin J, Guo A, Zhang H, Luo J, Gao G (2015). GSDS 2.0: an upgraded gene feature visualization server. Bioinformatics.

