# Supplementary material for: PHACTR1 splicing isoforms and eQTLs in atherosclerosis-relevant human cells
Source: BMC Med Genet. 2018 Jun 8;19:97. doi: 10.1186/s12881-018-0616-7 (PMC5994109; doi:10.1186/s12881-018-0616-7)
Supplement: Supplementary file 1 — List of primers used in this study. The numbers in the “Name” column refers to the primers shown in Fig. 1. All sequences are given in the 5′ to 3′ orientation. (PDF 43 kb) [file 12881_2018_616_MOESM1_ESM.pdf]

Additional file 1. List of primers used in this study. The numbers in the "Name" column refers to the primers shown in **Figure 1**. All sequences are given in the 5' to 3' orientation.

	Forward primers		Reverse primers	
	**Name**	**Sequence**	**Name**	**Sequence**
**RACE**	1	gattacgccaagcttGGACTCCCTAGAAAATGGGCAGTCCCTG	4	gattacgccaagcttGGCCCATCCATGATGTCTGACGGTTGG
	2	gattacgccaagcttGAGGAGGACGAAGACGACGACAGCTCATTA	5	gattacgccaagcttGGCCAGGGAGCTGGTGTATAATGAGCTG
	3	gattacgccaagcttGACCGCAGGGCAGATAAGCCGTGGACC		
	M13F	GTAAAACGACGGCCAGT	M13R	GGAAACAGCTATGACCATG
PacBio	dT-BC1	AAGCAGTGGTATCAACGCAGAGTACtcagacgatgcgtcatTTTTTTTTTTTTTTTTTTTTTTTTTTTTTTVN,		
	dT-BC2	AAGCAGTGGTATCAACGCAGAGTACctatacatgactctgcTTTTTTTTTTTTTTTTTTTTTTTTTTTTTTVN,		
	dT-BC3	AAGCAGTGGTATCAACGCAGAGTACtactagagtagcactcTTTTTTTTTTTTTTTTTTTTTTTTTTTTTTVN,		
	dT-BC4	AAGCAGTGGTATCAACGCAGAGTACtgtgtatcagtacatgTTTTTTTTTTTTTTTTTTTTTTTTTTTTTTVN,		
	dT-BC5	AAGCAGTGGTATCAACGCAGAGTACgatctctactatatgcTTTTTTTTTTTTTTTTTTTTTTTTTTTTTTVN,		
	dT-BC6	AAGCAGTGGTATCAACGCAGAGTACacagtctatactgctgTTTTTTTTTTTTTTTTTTTTTTTTTTTTTTVN		
RT-PCR	6	GCGGCATCCTCGGAGGATG	9	TCACTGGCAGACAAGGCAAT
	7	GGTTGCCTCCAATGTCAAGT	9	TCACTGGCAGACAAGGCAAT
	8	AGGTTTGCGCTCCGTTCG	9	TCACTGGCAGACAAGGCAAT
	10	ATGTATCTGCAAGGGCCGAG	11	CTCTTGATCTCTCTCTTCTCCTCCTG
qPCR	12	AGAGAGGCGGATGCATGTG	13	AGGGAGTCAGAACGCATCG
	8	AGGTTTGCGCTCCGTTCG	13	AGGGAGTCAGAACGCATCG
	14	ACCATGAAGAGACCCCAGTG	9	TCACTGGCAGACAAGGCAAT
	15	GGATGGGCCAGATCCTGGCG	16	CCTGCTGGCCACTCTTCTGG
	10	ATGTATCTGCAAGGGCCGAG	11	CTCTTGATCTCTCTCTTCTCCTCCTG
	TBP_F	CGAATATAATCCCAAGCGGTTT	TBP_R	GTGGTTCGTGGCTCTCTTATCC
	HPRT_F	TGGCGTCGTGATTAGTGATG	HPRT_R	CAGAGGGCTACAATGTGATGG
	GapDH_F	GACAGTCAGCCGCATCTTC	GapDH_R	GCAACAATATCCACTTTACCAGAG
