# Supplementary material for: PHACTR1 splicing isoforms and eQTLs in atherosclerosis-relevant human cells
Source: BMC Med Genet. 2018 Jun 8;19:97. doi: 10.1186/s12881-018-0616-7 (PMC5994109; doi:10.1186/s12881-018-0616-7)
Supplement: Supplementary file 2 — Genomic coordinates of exons based on the reference human genome sequence GRCh37/hg19. (PDF 47 kb) [file 12881_2018_616_MOESM2_ESM.pdf]

Additional file 2. GTF file. Genomic coordinates of exons based on the reference human genome sequence GRCh37/hg19.

seqname	source	feature	start	end	score	strand	frame	attribute
6	Ensembl	exon	12957636	12957776	.	+	.	transcript_id "with"; exon_id "06"
6	Ensembl	exon	12958214	12958269	.	+	.	transcript_id "with"; exon_id "07"
6	Ensembl	exon	13013852	13014371	.	+	.	transcript_id "with"; exon_id "78"
6	Ensembl	exon	13053597	13053761	.	+	.	transcript_id "with"; exon_id "08"
6	Ensembl	exon	13160436	13160516	.	+	.	transcript_id "with"; exon_id "09"
6	Ensembl	exon	13182751	13182918	.	+	.	transcript_id "with"; exon_id "10"
6	Ensembl	exon	13185031	13185237	.	+	.	transcript_id "with"; exon_id "1011"
6	Ensembl	exon	13206047	13206368	.	+	.	transcript_id "with"; exon_id "11"
6	Ensembl	exon	13228048	13228295	.	+	.	transcript_id "with"; exon_id "12"
6	Ensembl	exon	13230269	13230425	.	+	.	transcript_id "with"; exon_id "13"
6	Ensembl	exon	13273092	13273147	.	+	.	transcript_id "with"; exon_id "14"
6	Ensembl	exon	13278500	13278561	.	+	.	transcript_id "with"; exon_id "15"
6	Ensembl	exon	13283654	13283794	.	+	.	transcript_id "with"; exon_id "16"
6	Ensembl	exon	13286378	13286454	.	+	.	transcript_id "with"; exon_id "17"
6	Ensembl	exon	13287295	13288073	.	+	.	transcript_id "with"; exon_id "18"
6	Ensembl	exon	12957636	12957776	.	+	.	transcript_id "without"; exon_id "06"
6	Ensembl	exon	12958214	12958269	.	+	.	transcript_id "without"; exon_id "07"
6	Ensembl	exon	13013852	13014371	.	+	.	transcript_id "without"; exon_id "78"
6	Ensembl	exon	13053597	13053761	.	+	.	transcript_id "without"; exon_id "08"
6	Ensembl	exon	13160436	13160516	.	+	.	transcript_id "without"; exon_id "09"
6	Ensembl	exon	13182751	13182918	.	+	.	transcript_id "without"; exon_id "10"
6	Ensembl	exon	13206047	13206368	.	+	.	transcript_id "without"; exon_id "11"
6	Ensembl	exon	13228048	13228295	.	+	.	transcript_id "without"; exon_id "12"
6	Ensembl	exon	13230269	13230425	.	+	.	transcript_id "without"; exon_id "13"
6	Ensembl	exon	13273092	13273147	.	+	.	transcript_id "without"; exon_id "14"
6	Ensembl	exon	13278500	13278561	.	+	.	transcript_id "without"; exon_id "15"
6	Ensembl	exon	13283654	13283794	.	+	.	transcript_id "without"; exon_id "16"
6	Ensembl	exon	13286378	13286454	.	+	.	transcript_id "without"; exon_id "17"
6	Ensembl	exon	13287295	13288073	.	+	.	transcript_id "without"; exon_id "18"
6	Ensembl	exon	13272908	13273091	.	+	.	transcript_id "short"; exon_id "av14"
6	Ensembl	exon	13273092	13273147	.	+	.	transcript_id "short"; exon_id "14"
6	Ensembl	exon	13278500	13278561	.	+	.	transcript_id "short"; exon_id "15"
6	Ensembl	exon	13283654	13283794	.	+	.	transcript_id "short"; exon_id "16"
6	Ensembl	exon	13286378	13286454	.	+	.	transcript_id "short"; exon_id "17"
6	Ensembl	exon	13287295	13288073	.	+	.	transcript_id "short"; exon_id "18"
