# Supplementary material for: PHACTR1 splicing isoforms and eQTLs in atherosclerosis-relevant human cells
Source: BMC Med Genet. 2018 Jun 8;19:97. doi: 10.1186/s12881-018-0616-7 (PMC5994109; doi:10.1186/s12881-018-0616-7)

Additional file 3. Su mmary of observed transcripts with their alternatively spliced 5' and 3' untranslated regions (UTRs). The blue rectangles on the left indicate transcripts that were detected in tissue samples whereas gray rectangles mean they were not detected. Introns are not shown for simplicity; black lines indicate that the exons were not present in the isoform.

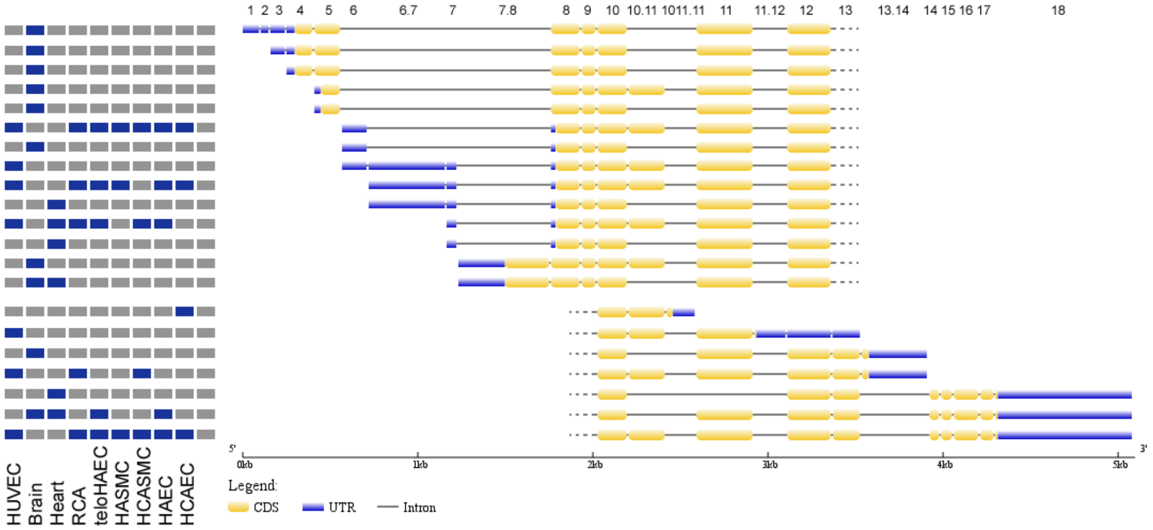

Supplement: Supplementary file 3 — Summary of observed transcripts with their alternatively spliced 5′ and 3′ untranslated regions (UTRs). The blue rectangles on the left indicate transcripts that were detected in tissue samples whereas gray rectangles mean they were not detected. Introns are not shown for simplicity; black lines indicate that the exons were not present in the transcript. (PDF 75 kb) [file 12881_2018_616_MOESM3_ESM.pdf]
