# Supplementary material for: PHACTR1 splicing isoforms and eQTLs in atherosclerosis-relevant human cells
Source: BMC Med Genet. 2018 Jun 8;19:97. doi: 10.1186/s12881-018-0616-7 (PMC5994109; doi:10.1186/s12881-018-0616-7)
Supplement: Supplementary file 4 — Transcript and protein IDs of PHACTR1 gene (221,692; ENSG00000112137). (PDF 45 kb) [file 12881_2018_616_MOESM4_ESM.pdf]

Additional file 4. T ranscript and protein IDs of *PHACTR1* gene (221692; ENSG00000112137).

	Length (bp)	Transcript IDs	Length (aa)	Protein IDs
Long	1743	NM_030948.3, ENST00000332995.11	580	NP_112210.1
Intermediate A-	1746	XM_017010464.1	581	XP_016865953.1
Intermediate A+	1953	NM_001322314.1	650	NP_001309243.1
Intermediate B-	1467	NM_001322312.1	488	NP_001309241.1
Intermediate B+	1674	NM_001322313.1	557	NP_001309242.1
Short	435	ENST00000379335.7	144	EAW55320.1
