# Supplementary material for: PHACTR1 splicing isoforms and eQTLs in atherosclerosis-relevant human cells
Source: BMC Med Genet. 2018 Jun 8;19:97. doi: 10.1186/s12881-018-0616-7 (PMC5994109; doi:10.1186/s12881-018-0616-7)

Additional file 5. Zoom-in representation of *PHACTR1* exon 14. The 5'-14 fraction of exon 14 is specific to the short immune-specific *PHACTR1* transcript. We also illustrate the 3' splice site used for the long and intermediate *PHACTR1* transcripts. In blue is the intron between exons 13 and 14. The red arrow indicates a primer used to detect the short *PHACTR1* transcript. The sequence of this primer #9 is in **Additional file 1**.

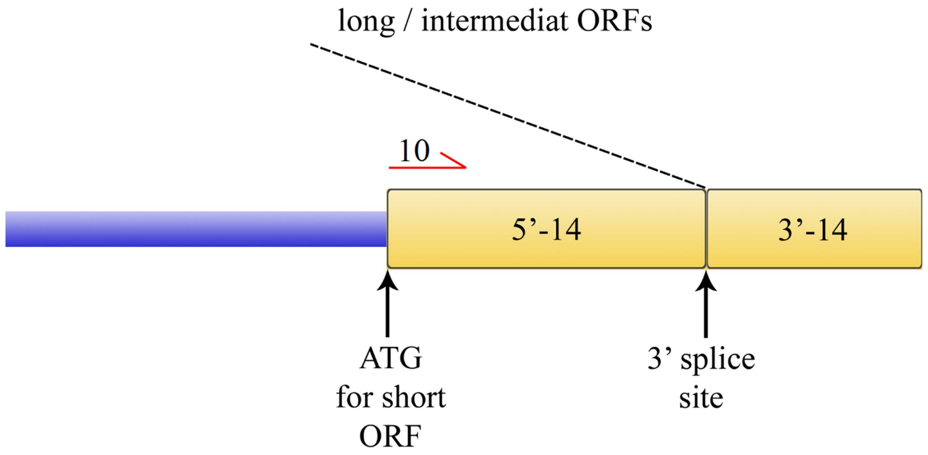

Supplement: Supplementary file 5 — Zoom-in representation of PHACTR1 exon 14. The 5′-14 fraction of exon 14 is specific to the short immune-specific PHACTR1 transcript. We also illustrate the 3′ splice site used for the long and intermediate PHACTR1 transcripts. In blue is the intron between exons 13 and 14. The red arrow indicates a primer used to detect the short PHACTR1 transcript. The sequence of this primer #10 is in Additional file 1. (PDF 193 kb) [file 12881_2018_616_MOESM5_ESM.pdf]
