# Supplementary material for: PHACTR1 splicing isoforms and eQTLs in atherosclerosis-relevant human cells
Source: BMC Med Genet. 2018 Jun 8;19:97. doi: 10.1186/s12881-018-0616-7 (PMC5994109; doi:10.1186/s12881-018-0616-7)

Additional file 6. Associations between genotypes at rs9349379 and *PHACTR1* expression levels in human coronary arteries. (**A**) By qPCR using transcript-specific primers, we measured the expression of *PHACTR1* transcripts in 36 human coronary arteries (hCA)(N_{AA}=15, N_{AG}=13, N_{GG}=8). The long *PHACTR1* transcript is not expressed in hCA. (**B**) We used GTEx data to test the associations between rs9349379 and the expression levels of *PHACTR1* exons in 122 hCA (N_{AA}=48, N_{AG}=57, N_{GG}=17). Here, we only show results for four exons, but association results for all *PHACTR1* exons are available in **Table 2**. Suppl. exon corresponds to the alternatively spliced exon located between exons 10 and 11. 5' exon 14 corresponds to part of exon 14 that is specific to the short *PHACTR1* transcript.

A

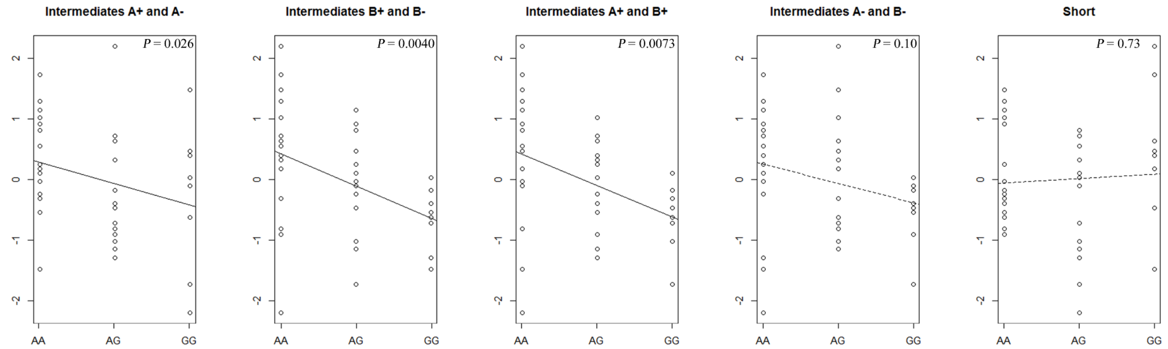

B

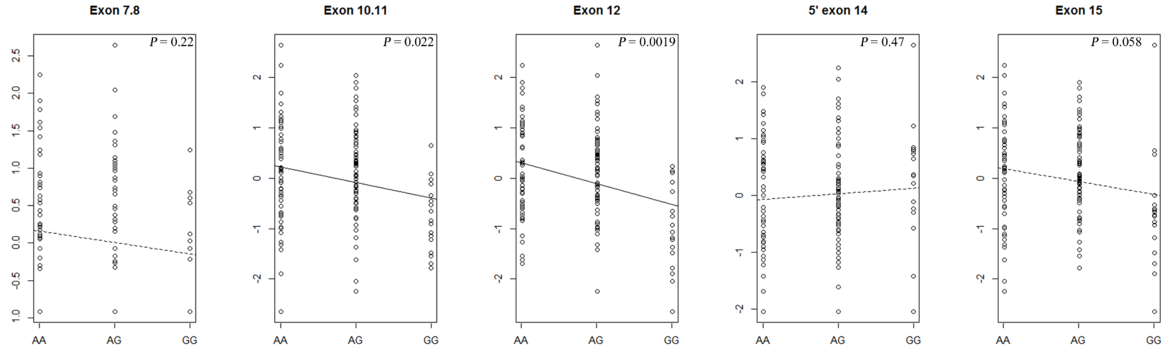

Supplement: Supplementary file 6 — Associations between genotypes at rs9349379 and PHACTR1 expression levels in human coronary arteries. (A) By qPCR using transcript-specific primers, we measured the expression of PHACTR1 transcripts in 36 human coronary arteries (hCA)(NAA = 15, NAG = 13, NGG = 8). The long PHACTR1 transcript is not expressed in hCA. (B) We used GTEx data to test the associations between rs9349379 and the expression levels of PHACTR1 exons in 122 hCA (NAA = 48, NAG = 57, NGG = 17). Here, we only show results for five exons, but association results for all PHACTR1 exons are available in Table 2. Exon 10.11 corresponds to the alternatively spliced exon located between exons 10 and 11. 5′ exon 14 corresponds to part of exon 14 that is specific to the short PHACTR1 transcript. (PDF 350 kb) [file 12881_2018_616_MOESM6_ESM.pdf]
